# Results of home-based modified combined decongestive therapy in patients with lower extremity lymphedema

**DOI:** 10.3906/sag-1809-25

**Published:** 2019-04-18

**Authors:** Alis KOSTANOĞLU, Meltem RAMOĞLU, Ethem GÜNEREN

**Affiliations:** 1 Department of Physiotherapy and Rehabilitation, Faculty of Health Sciences, Bezmiâlem Vakıf University, İstanbul Turkey; 2 Department of Plastic, Reconstructive, and Aesthetic Surgery, Faculty of Medicine, Bezmiâlem Vakıf University, İstanbul Turkey

**Keywords:** Lower extremity lymphedema, self-management, complex decongestive therapy

## Abstract

**Background/aim:**

The aim of this study is to present the results of modified combined decongestive therapy (CDT) in patients with lower extremity lymphedema (LEL).

**Materials and methods:**

We retrospectively reviewed 95 patients aged 55.84 ± 15.70 years who had been diagnosed with LEL between May 2015 and May 2017. The patients were treated for 4 weeks with modified CDT, including self-manual lymphatic drainage, self-bandaging, decongestive exercises, and skin care.

**Results:**

The mean reduction amounts of edema volume before and after treatment were 296.05, 784.92, and 1038.50 mL for stages 1, 2, and 3 respectively (P = 0.001). There were significant differences between the values before and after treatment in excess extremity volume (EEV) at all stages (P = 0.001). The EEV percentages of the secondary LEL patients were higher than those of the primary LEL patients (P = 0.04). There was no correlation between BMI and treatment response in terms of EEV percentages (r = –0.99; P = 0.36).

**Conclusion:**

Our results revealed that home-based modified CDT is more effective in reducing extremity edema volume in secondary LEL than primary LEL. It should be an available method for self-management of LEL at all stages.

## 1. Introduction

Lymphedema is a chronic, progressive condition that can affect a significant number of people and have effects on patients’ physical and physicosocial health [1]. A substantial number of patients suffer from this irreversible, permanent, and debilitating disease. Without proper management, lymphedema may progress, resulting in the proliferation of fibrotic tissue, an increase in the size of the affected extremity, and increased risk for wounds and infections [2,3]. Lower extremity lymphedema (LEL) may occur as a primary (dysfunction of the lymphatic system) or secondary (e.g., removal of lymph nodes, radiation therapy, trauma) condition [4,5].

LEL management is life-long, and it may include mainly conservative, sometimes medical, and rarely surgical treatments [6–9]. The gold standard treatment of lymphedema is combined decongestive therapy (CDT) [10,11]. CDT consists of a two-step program carried out by a professional team, including specifically trained lymphologists and physiotherapists. The first step is known as Phase 1, which primarily includes skin care, manual lymphatic drainage (MLD), use of multilayered compression bandages, and exercises. The second step, called Phase 2, is primarily seen as a maintenance stage and comprises skin care, self-MLD, use of a compression stocking, and exercises [12,13]. 

Although it is effective, CDT is criticized for being time-consuming, costly, and insufficiently researched [14]. It requires trained health professionals, can be difficult for the patient to access, and is expensive as it is provided in an outpatient setting, but early intervention and good self-management can prevent progression of symptoms. With proper compliance and thorough instruction by the clinician, the majority of patients are able to maintain and improve the treatment results achieved during the intensive phase of the therapy. Healthcare professionals often teach patients or their caregivers a simplified version of MLD and self-bandaging [13,14]. Successful management of lymphedema relies on patients and caregivers to play an active role in the process. 

According to our literature review, there are few papers on the results of home-based CDT in patients with LEL. The aim of this study is to present the practicality and ease of treatment modification, as well as the results experienced by our patients.

## 2. Materials and methods

Medical chart reviews of 159 LEL patients who were consecutively treated in the Department of Plastic, Reconstructive, and Aesthetic Surgery at Bezmiâlem Vakıf University between July 2015 and June 2017 were evaluated retrospectively. The institutional ethics committee approved this study, which was performed in accordance with the ethical principles for human research as outlined by the Declaration of Helsinki, Second Revision.

### 2.1. Participants

We chose patients for the treatment of modified-CDT from amongst those who had already received CDT but failed due to financial reasons or transportation, and those who previously had unsatisfactory results from CDT and wanted to try self-management.

Thirty-one patients had bilateral LEL, 3 patients had genital lymphedema, and 30 patients were recommended only compression garments. Ninety-five patients were found to have LEL. The inclusion criteria were as follows: the patient must be older than 18 years of age, must have suffered from primary or secondary unilateral lower extremity lymphedema, and must have previously received CDT treatment. The exclusion criteria were: having a metastatic disease; having a previous diagnosis of coronary heart disease, pulmonary disease, or an acute infection for any reason; lower extremity peripheral arterial occlusive diseases; or peripheral neuropathy. The clinical stages of lymphedema for our cases were classified based on the International Society of Lymphology [6]. Body mass index (BMI) was calculated by dividing weight in kilograms by the square of height in meters. BMIs of between 18.5 and 24.9, between 25 and 29.9, and higher than 30 were defined as normal weight, overweight, and obese, respectively.

### 2.2. Measurements

The circumferences of the extremities were measured when the patients were placed in a long sitting position, with the ankle in the neutral position, from the first metatarsophalangeal joint to the proximal with 4-cm intervals for each. The values that were obtained were recorded in centimeters and converted to extremity volume using Kuhnke’s disc method. The circumference measurements were repeated before and after treatment in all patients [15]. 

The therapeutic response of modified CDT was quantified as the change in the percentage of excess extremity volume (EEV) using the following formula: EEV (%) = (pretreatment volume – posttreatment volume/posttreatment volume) × 100.

### 2.3. Treatment method

The patients at our outpatient clinic received a basic introduction to skin care and risk reduction training. Modified CDT consisted of self-MLD, self-bandaging, and decongestive exercises training. The patients were clinically followed once a week for 4 weeks under the supervision of a trained physiotherapist. 

The first session started with one-on-one teaching with detailed explanation of the theory and principals of an effective therapy program. A trained physiotherapist demonstrated the self-MLD technique while the caregiver videotaped it. In self-MLD, to stimulate lymph drainage, the patient performed simple MLD techniques at least once a day for 10–15 min. Stationary circles were used, and the patients executed this technique using light pressure.

In the second step, the physiotherapist demonstrated the self-bandaging technique in a sequence of steps, using a diagram to aid comprehension. The caregiver was allowed to apply this technique immediately in this session, and it was videotaped while it was being applied by the physiotherapist. The patients were trained on the warning signs and symptoms of inappropriate application of bandages, including pain, discomfort, tingling, or numbness of fingers. They were advised to remove the bandages completely and reapply. If symptoms and signs persisted, they were asked to skip bandages and apply other methods of treatment until the next week’s hospital visit. Short stretch bandages have limited extensibility under tension (50%). Multilayer, multicomponent, and short stretch bandages consisted of a cotton padding layer, a short stretch cohesive bandage (Mollelast Haft), and a short stretch nonadhesive bandage (Rosidal K) on the top (Lohmann & Rauscher, Rengsdorf, Germany). The three components were applied in a spiral style, with 50% of overlap between the layers, from the base of the toes up to 2 cm below the hip. Mollelast Haft and Rosidal K were applied under full stretch to exert a strong-to-very strong pressure according to the International Compression Club’s classification of compression materials. The participants were told to keep the bandages 7 days a week for 16 h a day on average. The bandages were removed while taking a shower and at night, and then reapplied in the morning. When they felt discomfort, they were asked to remove the bandages and to note this in their logbooks.

For decongestive exercises, breathing exercises, gluteal sets, knee sets, and toe flexion-extension exercises were performed for 10–15 min following the self-bandaging session. The exercises were performed while lying in a supine position. The patients would rest with the extremity elevated for 10–15 min after the exercises. The exercises were shown to the patients individually during this session and booklets with visual and written instructions were distributed.

For skin care, appropriate skin moisturizers should be applied twice a day to maintain the health and integrity of the skin. The patients were informed about skin care and protection of the integrity of the skin. A booklet containing information about what should not be done and potential situations the patients should protect themselves from was distributed. 

The patients, who visited the outpatient clinic every week for 4 weeks, were assessed weekly for extremity volume measurements. The videotape was reviewed when the patient returned to the clinic, and recommendations for corrections in self-treatment were made based on the video content.

### 2.4. Statistical analysis

Statistical analysis was performed using SPSS 21.0 for Windows (IBM Corp., Armonk, NY, USA). Kruskal–Wallis analysis of variance (post hoc Tukey HSD test) was used to evaluate the differences in the changes between the stages of LEL in all parameters. The nonparametric Wilcoxon matched pairs test was used for intragroup comparison of the results before and after treatment. The Mann–Whitney U test was applied to determine the relationship between the reduction in EEV percentage and BMI, age, and duration of lymphedema. The Tamhane T2 test was used to determine the 3 groups’ mean differences of EEV% because of the groups’ unequal variance and sample size. Statistical significance was accepted at P < 0.05.

## 3. Results

The study included 95 patients aged 55.84 ± 15.70 years; 34 (35.79%) were female and 61 were male (64.21%). The mean body mass index was 31.21 ± 7.36 kg/m2. The demographic characteristics of the patients are shown in Table 1. The distribution of the patients based on the affected extremity was as follows: 40 (42.11%) left extremity, 55 (57.89%) right extremity. The patients were diagnosed as having either primary (n = 24, 25.26%) or secondary (n = 71, 74.74%) LEL. The lymphedema severity in the patients was evaluated and classified as stage 1 (mild) (32.63%), stage 2 (moderate) (50.53%), or stage 3 (severe) (16.84%). The mean duration of lymphedema was 36 months (range: 1–600). The beginning of lymphedema was 0–6 months ago for 15 patients (15.79%), 7–12 months ago for 10 (10.53%), 13–24 months ago for 14 (14.74%), 25–60 months ago for 33 (34.74%), and earlier than 60 months ago for 23 (24.71%). Other comorbidities of the patients were also investigated.

**Table 1:** Characteristics of the patients’ lower extremity lymphedema.

	N	%
Age (years)18–3031–5051–65Over 65	7293227	7.3730.5333.6828.42
SexFemaleMale	3461	35.7964.21
Body mass index (kg/m2) (mean ± SD)	31.21 ± 7.36	
Dominant extremityLeftRight	1184	11.5888.42
DiagnosisPrimary lymphedemaSecondary lymphedema	2471	25.2674.74
Etiologies of secondary LEL PhlebolymphedemaLipolymphedemaMelanomaPostinfectiousGynecologic cancers	281215511	29.4712.6315.795.2611.58
Affected extremity Left Right	4055	42.1157.89
Lymphedema severityStage 1 (mild)Stage 2 (moderate)Stage 3 (severe)	314816	32.6350.5316.84
Duration of lymphedema (months) (min–max)	36 (1–600)	

LEL = Lower extremity lymphedema.

Differences of extremity edema volume before and after treatment were classified based on the stages of lymphedema (Table 2). There were significant differences before and after treatment at all stages (P < 0.001). The mean reduction amounts of edema volume before and after treatment were 296.05, 784.92, and 1038.50 mL for stages 1, 2, and 3 respectively. There was a correlation between stage 1 and stages 2 and 3 (r = 0.067; P < 0.001). There was no correlation between stage 2 and stage 3 (r = 0.070; P > 0.05). The groups were divided based on cases of primary and secondary lymphedema to compare BMI, age, lymphedema duration, and EEV percentage (Table 3). There was a statistically significant difference only for EEV percentage (P = 0.40). The EEV percentages of the secondary LEL patients were higher than those of the primary LEL patients. The correlation results between BMI and treatment response in terms of EEV percentage (r = –0.99; P = 0.36) are shown in Table 4.

**Table 2:** Comparison of pre- and posttreatment extremity volumes based on stages.

	V0*Mean ± SD	V1**Mean ± SD	V0-V1***Mean ± SD	P
Stage 1 (mL)	5510.88 ± 2308.41	5214.83 ± 2200.40	296.05 ± 232.88	0.001
Stage 2 (mL)	6853.50 ± 2522.73	6068 ± 1820.90	784.92 ± 868.24	0.001
Stage 3 (mL)	7736.60 ± 2985.23	6698.10 ± 2772.16	1038.50 ± 553.06	0.001

* Initial extremity volume. ** Posttreatment extremity volume. *** Difference between initial and posttreatment extremity volumes.

**Table 3:** Comparison of primary and secondary lower extremity lymphedema in terms of age, BMI, and EEV percentage.

	Primary Mean (min–max)	Secondary Mean (min–max)	P
Age (years)	56.5 (18–80)	56 (24–89)	0.06
BMI (kg/m2)	30 (20–53)	30 (17–49)	0.07
EEV %	6 (1–22)	9 (1–28)	0.04

BMI = Body mass index; EEV = excess extremity volume.

**Table 4:** Comparison of mean difference of EEV% among BMI groups.

BMI Groups		Mean difference of EEV % Mean ± SD	P
Normal	Overweight	–1.72 ± 1.53	0.60
	Obese	–0.57 ± 1.52	0.97
Overweight	Normal	1.72 ± 1.53	0.60
	Obese	1.14 ± 1.38	0.79
Obese	Normal	0.57 ± 1.52	0.97
	Overweight	–1.14 ± 1.38	0.79

BMI: Body mass index; EEV%: excess extremity volume percentage.

## 4. Discussion

LEL is a chronic debilitating disease that requires life-long management. Today, the gold standard of treatment for lymphedema is CDT [6]. The aim of this study was to determine the effects of home-based modified CDT on LEL. Our results revealed that home-based modified CDT had positive effects on volume reduction in patients at all stages of LEL. The EEV percentage in patients with secondary lymphedema was higher than that in the primary lymphedema patients.

In the literature, several studies highlighted the distinct lack of evidence for the optimal management of lymphedema [16]. Although effective, clinically administered CDT is criticized as being time-consuming, costly, and insufficiently researched [17], failure to continue treatment may result in an increase in swelling, infections, and pain and have a negative effect on the patient’s self-image. Supporting patient self-management is a key component of effective chronic illness care and improved patient outcome [18]. The patient’s involvement in their own treatment is a part of self-management programs. Clark et al. [19] suggested that individuals also have to cope with the psychosocial problems generated by their chronic disease and must manage their daily lives based on their financial and social statuses. 

Previous studies of risk factors for the development of LEL have been limited and investigated the roles of BMI [20]. Graf et al. [21] revealed that time of improvement in LEL decreased with the BMI of the patient, considering that weight gain and higher BMI are risk factors for development and increased severity of lymphedema. According to the results of our study, as BMI increases, there might be a threshold above which lymphatic flow becomes impaired. Proximal transport of lymphatic fluid from the extremity is dependent on the function of the lymphatic vasculature and the volume of lymph produced by the tissues. As the amount of adipose tissue increases in the lower extremities, lymphatic vessels may become dysfunctional, thereby reducing proximal lymphatic flow. Besides, a higher BMI increases complication rates, whereas a lower BMI provides a significant reduction in overall survival and complication rates [22]. Underweight patients (BMI < 18.5 kg/m2) with locally advanced cervical cancer have lower overall survival rates and more complications than normal-weight and obese patients [23]. The results of our study showed that there was a decrease in the percentage of EEV, but this decrease was not statistically significant. Studies in the literature have focused on the results of secondary LEL development related to high BMI. Our sample included both primary and secondary LEL patients.

Regardless of the type of volume-reduction treatment, burdensome lifelong self-care that includes compression, self-administered MLD, and skin care is required to maintain volume reduction after CDT or achieve additional volume reduction. Zhang et al. [24] compared breast cancer patients scheduled for modified radical mastectomy who were randomly apportioned to undergo physical exercise only (n = 500) or self-MLD as well as exercise (n = 500) after surgery. The results of their study indicated that self-MLD, in combination with physical exercise, is beneficial for breast cancer patients in preventing postmastectomy scar formation, upper limb lymphedema, and shoulder joint dysfunction. Bernhard et al. [25] compared the effects of complex decongestive physiotherapy carried out either by a physiotherapist or by patients themselves under the supervision of the physiotherapist. Forty-six patients were treated and followed for 9 months. Significant reductions were recorded after 3, 6, and 9 months of performing comprehensive self-treatment including compression therapy (n = 33 legs). The patients in this study consisted only of lymphatic filariasis patients. Although our treatment results obtained higher EEV percentages in secondary LEL patients, they also showed that treatment was effective in primary LEL patients. Another study assessed the quality of life in participants performing self-lymphatic drainage with or without aromatherapy, and both groups reported significant improvements at all time points up to 6 months [26]. Assigning more responsibility to patients in handling lymph drainage and bandaging resulted in a reduction in leg volume. Following initial treatment, adherence to the program and follow-up were limited by the impracticality of compressive stockings and their tendency to deteriorate rapidly due to harsh environments. Our sample consisted of patients who had started treatment before but could not continue due to reasons of health insurance, transportation, or financial resources. All patients had previously received CDT including MLD and compression bandages. The maintenance phase was unfinished as some of the patients could not use the compression stockings proposed in the maintenance phase. The fact that they were contributing to their own treatments with the modified CDT method at home may have caused them to feel good about themselves. None of them left their treatment or follow-up procedures. 

In a previous study, a case series about self-bandaging, 30 upper and lower extremity lymphedema patients received a self-bandaging training program during 3–12 weeks of treatment [27]. Among all participants with LEL, edema reduction after intensive self-bandaging showed statistically significant differences at all stages. In our results, there were no statistically significant differences between mild and severe lymphedema. Sherman and Koelmeyer [28] showed a link between perceived self-regulation, self-efficacy, and lymphedema risk reduction behaviors. Success of self-bandaging in reducing the amount of edema may bring an increased sense of independence and a resulting feeling of being in control of the situation. The self-bandaging program had to be monitored by professionals, and corrections were made in the technique or administration performed by the patient or their caregiver(s). In our study, patients recorded a video of both self-MLD and self-bandaging. We used this method to overcome treatment barriers like transportation problems, coordinating appointment times with the patient, and the patients’ mobility problems. The video review/correction method helps provide an educational component to home-based self-management of LEL. In a study that used a tele-rehabilitation program, including sharing educational videos with LEL patients, a viable method was found to eliminate potential physical treatment barriers [29].

There are several limitations of this study. The results of this study reflected only 4 weeks of modified CDT implementation. Because of the lack of long-term follow-up evaluations, we could not comment on protection of benefits. Thus, only the volume reduction rates of the patients were provided, and whether or not actual physical improvements of the limb(s) occurred is unknown. There was only one intervention group. Despite the limitations, the study obtained valuable information for further studies.

In conclusion, we retrospectively reviewed the results of home-based modified CDT treatments. The findings of our study revealed the effects of home-based modified CDT on patients who had received treatment before but failed in the maintenance phase. This topic should be examined by randomized controlled studies in large samples, and the procedure’s safety should be determined and applied. Our results revealed that home-based modified CDT is more effective in reducing extremity edema volume in secondary LEL than primary LEL. It should be an available method for self-management of LEL at all stages.
